# Leveraging image processing techniques to visualize sub-cellular domains in optical photothermal infrared imaging

**DOI:** 10.1039/d6an00207b

**Published:** 2026-07-16

**Authors:** Elisabeth Holub, Nikolaus Hondl, Margaux Petay, Sarah Reindl, Sophie Honeder, Tamara Tomin, Bernhard Lendl, Georg Ramer

**Affiliations:** a TU Wien, Institute of Chemical Technologies and Analytics Getreidemarkt 9 1060 Wien Austria; b TU Wien, Christian Doppler Laboratory for Advanced Mid-Infrared Laser Spectroscopy in (Bio-)process Analytics Getreidemarkt 9 1060 Wien Austria georg.ramer@tuwien.ac.at +43 1 58801 164151

## Abstract

Traditionally, vibrational spectroscopic imaging captures a full spectrum at every pixel, a technique commonly referred to as hyperspectral imaging. With the emergence of high-resolution vibrational imaging methods such as optical photothermal infrared (O-PTIR) spectroscopy, it has become a widespread practice to perform imaging at specific wavenumber settings instead of acquiring full hyperspectral data cubes to speed up the acquisition process. Yet, regardless of the dimensionality of the data, spectroscopic imaging modalities require elaborate data pre-processing to reduce background noise, scattering effects, and imaging artifacts that may otherwise distort chemical information and prevent the reliable implementation of feature extraction and classification algorithms. Although the spectral analysis of chemical images has been extensively studied, there is limited research on spatial pre-processing techniques such as masking and spatial filtering, which may become more important when images are acquired at select wavenumbers only. Furthermore, processing pipelines are often vendor-specific, hampering the comparability of research results, or rely on bespoke algorithms and non-standard dependencies, leading to long-term maintenance and compatibility issues. The aim of this paper is hence to present a straightforward, step-by-step protocol for the spatial processing and subsequent segmentation of sparse chemical imaging data using standard open-source libraries. We demonstrate our approach on a set of single-wavenumber images of human cells recorded with a home-built O-PTIR instrument.

## Abstract 2

O-PTIR imaging is performed at multiple wavenumbers on a human cell. After pre-processing and segmentation, an overlay image is produced, in which different colors indicate the relative contribution of the individual components.

## Graphical abstract

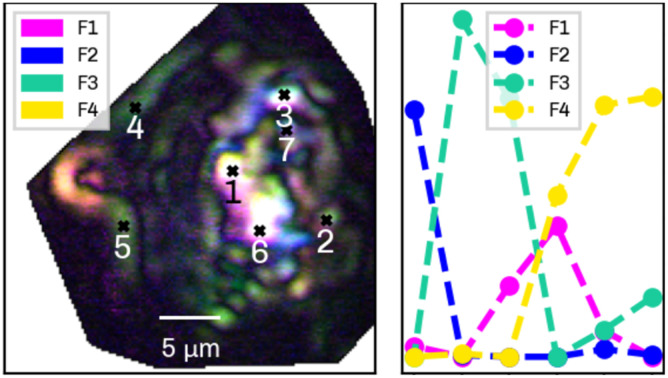

## 1. Introduction

Detecting and visualizing minute structural variations beyond morphological characteristics is a crucial task in the analysis of biomedical samples. The technique commonly used to investigate biological specimens *in vitro* and *in vivo* is fluorescence microscopy, which has become a standard tool in life science imaging^1^ and clinical diagnosis.^2^ Fluorescence imaging relies on the use of labeling agents, which can potentially degrade the viability of the sample and influence its intrinsic properties.^3,4^ In addition, the fluorescence signal does not necessarily correspond to the sample region of interest^5^ and suffers from autofluorescence.^4^ Therefore, label-free imaging technologies are constantly sought to circumvent these issues.

Offering both stain-free chemical analysis and spatially resolved information, vibrational imaging modalities such as Raman microscopy and Fourier Transform Infrared (FTIR) spectroscopic imaging have been increasingly used in biomedical applications such as the identification of cancer markers,^6^ the analysis of sub-cellular structures and metabolic activities of cells,^7–9^ histopathological analysis of tissue sections,^10–13^ and early detection of disease.^14,15^ Despite their powerful capabilities for chemical imaging, Raman and FTIR spectroscopy have inherent limitations. The Raman cross section is very small compared to the IR absorption cross section of biological samples,^16^ thus affecting the sensitivity of the technique. In a similar way, infrared (IR) microscopy suffers from low spatial resolution due to the physical limit of diffraction, which dictates that the minimum resolvable distance increases with the wavelength of the light source. While recent technological advancements, most notably the replacement of thermal light sources with QCLs,^17^ have improved signal-to-noise ratio and image acquisition speed, the fundamental limit of diffraction persists.

Bridging the gap between the high chemical sensitivity and specificity of IR spectroscopy and the sub-micrometer spatial resolution of light microscopy, a hybrid method termed optical photothermal infrared (O-PTIR) spectroscopy has been proposed.^18^ Originally developed in the 1980s,^19,20^ O-PTIR spectroscopy leverages site-specific changes in the refractive index upon the absorption of radiation to analyze the chemical properties of a sample. Since the refractive index of a material changes with temperature, sample molecules irradiated by a pump beam will create a refractive index profile which can be detected by a second laser beam. Using a visible probe laser, O-PTIR microscopes can overcome the diffraction limit of traditional infrared imaging techniques to achieve sub-micrometer resolution.^21,22^ Exploiting the broad tuning range and monochromatic emission provided by quantum cascade lasers (QCLs), O-PTIR spectroscopy and imaging allow for the highly sensitive and label-free detection of cell and tissue constituents^23,24^ and offer a huge potential for characterizing biomedical samples, computational staining, and chemical imaging. Although photothermal infrared spectroscopy has been used and studied for decades, its application to imaging is a rather recent development and has led to a surge in research efforts in the field. Examples from life sciences and medicine include hydrated tissue sections,^24^ amyloid plaques in brain tissue,^25^ live cells,^23^ and lymph nodes.^26^

One of the main challenges of vibrational spectroscopic imaging is that raw spectral data cannot be interpreted directly due to substantial variations in background noise and instrument responses, pixel defects in detector arrays, and artifacts inherent to the technique employed.^27–29^ Hence, image pre-processing is an indispensable step to ensure the comparability and consistency of data, and consequently the robust analysis of measurements across different instruments and settings.

As vibrational images typically oversample in the spectral and/or the spatial domain, the resulting datasets have high dimensionality as well as considerable correlation and redundancy, making high-throughput analysis difficult and biasing feature extraction and classification.^13,28,30–32^ To meet the need to reduce the number of spectral descriptors and noise, dimensionality reduction techniques such as principle component analysis (PCA) and filter methods have been extensively explored.^8,14,33,34^ At the same time, approaches using only part of the spectrum or even discrete spectral bands have been on the rise and have demonstrated that a set of distinct wavenumbers may be sufficient for classification and compositional analysis.^30,35–38^

Numerous tutorials and user guides have been written for the analysis of chemical and hyperspectral images.^27,28,34,39,40^ However, their applicability to discrete-wavenumber O-PTIR microscopy is limited, in particular when it comes to life science applications, which are characterized by high sample complexity and variability on the one hand and low throughput capabilities on the other. This limitation stems from the fact that, while O-PTIR spectroscopy probes vibrational transitions just like traditional techniques such as FTIR and Raman spectroscopy, the signal generation and detection mechanisms differ significantly. Hence, baseline and artifact corrections must be tailored to each technique, focusing on detector drift and thickness in FTIR, fluorescence in Raman, and photothermal dynamics in O-PTIR spectroscopy.

Furthermore, there is a lack of detailed instructions as to the exact implementation and parameters of data processing scripts, which is attributable to the predominant use of proprietary software. Vendor-specific routines are often used in a “black-box” manner and have made it difficult to compare and reproduce results across imaging modalities and disciplines. In addition, hyperspectral imaging guides focus on spectral correction techniques such as baseline correction and spectral smoothing, but neglect spatial correction approaches like masking, which may become more important when images are recorded at a few distinct wavenumbers only.

To combine spatial and spectral features, convolutional neural network (CNN) architectures such as UNet^41^ have been suggested and are now available on open-source platforms. Despite their proven effectiveness in the segmentation of hyperspectral images, CNNs have the drawback of needing a large amount of training images, which are not readily available in high-resolution biomedical imaging. Moreover, the performance of a CNN model depends heavily on the optimization of hyperparameters such as the configuration and number of layers.

Against this background, our aim is to streamline the pre-processing and segmentation of multi-wavenumber chemical microscopy images by providing a detailed description of open-source routines that are applicable to a broad range of biomedical applications and instruments. The proposed workflow is specifically intended for scenarios in which the number of measurements is limited and reference measurements and/or standards are unavailable. The primary objective of this work is to provide a workflow based on open-source software for processing and analyzing sparse spectral imaging data while minimizing the influence of instrumental noise, unwanted scattering, and non-informative background contributions. In contrast to other methods, which require meticulous fine-tuning, the implemented routines have few or no hyperparameters and provide an output that is easier to interpret than the raw data.

After establishing a pre-processing pipeline, we will discuss the use of PCA, nonnegative matrix factorization (NMF) and k-means clustering to explore compositional patterns in chemical maps of individual lung cancer cells, which were acquired by a home-built O-PTIR instrument. In addition to chemical images, spectra were collected at selected points of interest to underpin the validity of the proposed image processing approach and to show the local character of the collected information. We demonstrate that local differences in cell composition can be detected and visualized using only a few specific wavenumber settings.

## 2. Materials and methods

### 2.1. Samples

H1299 cells were purchased from American Type Culture Collection (ATCC, Manassas, VA, USA) and cultured in RPMI 1640 (R0883, Sigma Aldrich), supplemented with 10% fetal bovine serum (F7524; Sigma Aldrich), and 2 mM glutamine (G7513, Sigma Aldrich). Approximately 250 000 cells were plated onto CaF_2_ glass (Crystran Ltd, Poole, UK) of 13 mm diameter and 1 mm thickness, placed in the wells of a 6-well plate, and the cells were allowed to adhere and grow for 48 hours. Thereafter, cells were fixed with 4% paraformaldehyde (PFA) in phosphate-buffered saline (PBS) buffer for 15 minutes. During fixation, cells were maintained at 37 °C to preserve cellular morphology. After fixation, the cells were stored in PBS buffer. Before analysis, PBS buffer supernatant was decanted, and the samples were rinsed twice with distilled water to remove excess PBS and prevent salt crystal formation.

The authors acknowledge that chemical fixation and subsequent rinsing can induce subtle alterations in cellular spectral signatures.^42^ However, the objective of this work is to reveal and visualize chemically distinct regions rather than to establish the absolute biochemical composition of the samples. Consequently, validation of fixation- or rinsing-induced spectral changes are beyond the scope of this study. All samples were prepared using the same fixation and rinsing protocol; therefore, any preparation-related spectral effects were common across the dataset and do not affect the methodology presented here.

### 2.2. O-PTIR instrument

O-PTIR spectro-microscopy was performed using a home-built confocal instrument described elsewhere.^35,43^ A tunable IR pump laser and a continuous visible probe laser are arranged in a counter-propagating geometry to detect the wavenumber-specific absorption of infrared (IR) radiation in the sample. The modulated IR laser induces a modulated local refractive index change proportional to the absorption of IR laser power by the sample. The photothermal signal is extracted from the intensity of the visible (633 nm) laser, which is altered by the fluctuating refractive index profile. Using high modulation frequencies, low-frequency noise sources are suppressed, and the photothermal signal can be recovered by a lock-in amplifier.

The spectral resolution, duty cycle and modulation frequency of the IR laser were set to 1 cm^−1^, 2.5%, and 50 kHz, respectively. The visible laser power was approximately 0.5 mW at the sample. The lateral spatial resolution of the home-built O-PTIR system is 615 nm ± 26 nm at an excitation wavenumber of 1490 cm^−1^ as previously established by Holub *et al.*^43^ In this prior work, the spatial resolution of the home-built system was studied using polystyrene beads. Since the relevant experimental conditions and laser settings were unchanged from the previous study, spatial resolution can be expected to remain consistent. Spectral resolution is 1 cm^−1^ as specified by the IR laser manufacturer. To remove atmospheric water vapor, the IR beam path was flushed with dry air. The fidelity and reproducibility of spectral features recorded with the O-PTIR prototype has been demonstrated in a prior study.^35^

### 2.3. Data collection and processing

All processing and data formatting operations were carried out using Python 3, in particular the NumPy^44,45^ and Xarray^46^ packages as well as the scikit-learn^47^ and scikit-image^48^ libraries. Baseline correction was performed with the open source package of pybaselines.^49^

#### 2.3.1. Chemical images

Chemical images were recorded at several wavenumbers to probe different absorption bands relevant for biological samples: the symmetric (*ν*PO_2_^−^, ≈1080 cm^−1^) and asymmetric (*ν*_as_PO_2_^−^, ≈1240 cm^−1^ (ref. 50)) stretching modes of the phosphodiester moiety, the Amide-II and Amide-I bands (with maxima at 1550 cm^−1^ and 1650 cm^−1^ (ref. 51)), and the C=O stretching vibration of triglycerides (≈1725 cm^−1^ to 1745 cm^−1^ (ref. 52)). The following wavenumbers were probed: 1100 cm^−1^, 1131 cm^−1^, 1236 cm^−1^, 1540 cm^−1^, 1660 cm^−1^, and 1740 cm^−1^. Target wavenumbers were identified through preliminary spectral sweeps, selecting values near the peak maxima that yielded the best signal-to-noise ratio (SNR) for high-resolution mapping. The wavenumber at 1236 cm^−1^ had emerged as a marker in a previous study on human cancer cells.^35^

The first step in image processing is the elimination of pixels that do not carry information. The largest subset of such pixels consists of those that lie outside the sample on the substrate. The photothermal signal is the difference in the transmitted laser intensity with and without pumping by the IR laser. For this reason, three signals are recorded per pixel: the in-phase and out-of-phase signals (extracted by a lock-in amplifier) plus the transmission signal, which is also used for normalization. One should note that varying sample thickness and topography can lead to a mismatch between the axial laser focus and the region of maximum photothermal effect. This is an inherent limitation of the technique that has been neglected in this work; however, the focus mismatch is typically outweighed by IR absorption and is therefore expected not to significantly compromise the overall findings.

The transmission image served as a starting point to compute a mask that would separate the sample from the background. Such a mask is easily obtained through the convex hull of a filtered image, that is, the smallest convex polygon enclosing all non-zero points. As even single pixels of elevated signal intensity will be included in the convex hull, it may be useful to correct for isolated pixels before computing the mask to make sure that only pixels within the desired sample region contribute to the analysis. Where a transmission image is unavailable, a reference brightfield image can be used instead. Furthermore, the signal intensities at wavelengths that are absorbed by the whole sample, *e.g.* the Amide I and II bands, are good candidates.

Several filters (see SI S2) were tested on the transmission image to obtain the region of interest (ROI) that would best represent the pixels containing the cell sample. A suitable filter would suppress as much background as possible without clipping the cell area. To obtain a sharp separation between two regions, thresholding filters are the obvious choice. The most promising results were achieved using Otsu,^53^ Li,^54^ and Sauvola^55^ filters. While Otsu's method maximizes the variance between the foreground and background pixel classes, Li's filter minimizes the cross-entropy between the foreground and the foreground mean, and the background and the background mean. Instead of finding a single threshold for the entire image, the Sauvola filter calculates local thresholds within a pre-defined window.

The filtered image was then converted to a binary image, from which the convex hull was computed. Isolated pixels were removed from the filtered image to keep the hull as close as possible to the actual sample region. The last step was to apply the binary mask to the chemical image. The established workflow was applied to different cells in different fields of view.

**Fig. 1:**
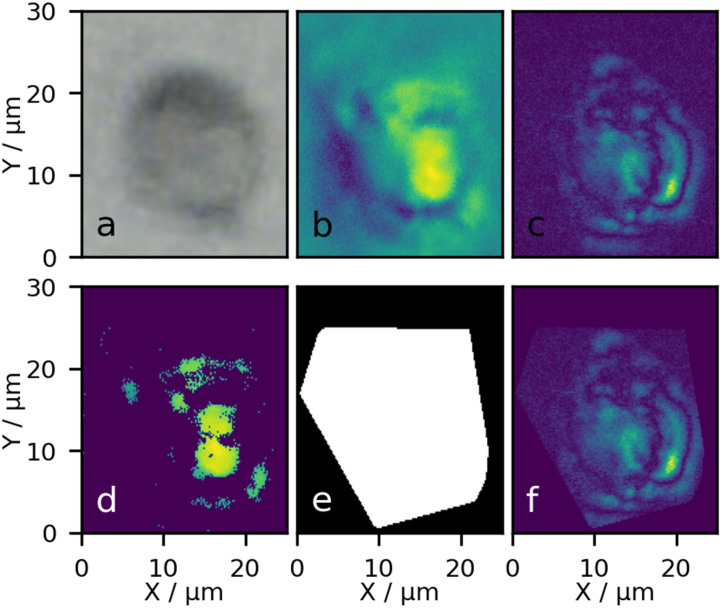
Image pre-processing procedure. (a) Brightfield image; (b) transmission image; (c) chemical image recorded at 1100 cm^−1^; (d) Sauvola filter applied to the transmission image; (e) resulting binary hull; (f) segmentation into ROI and background.

Fig. 1 illustrates the steps of the masking procedure. A transmission image (b) and an O-PTIR image (c) are acquired simultaneously. The transmission image is filtered (d); for the image presented, the Sauvola filter resulted in the best compromise between background suppression and avoiding cuts to the sample region. The filtered transmission image is used to generate a mask (e). The resulting image segmentation into ROI and background is shown in (f); a brightfield image (a) is provided for comparison.

#### 2.3.2. Analysis of sub-cellular domains

After segmentation into foreground and background, the individual chemical images were normalized by calculating the unit vector norm over each single-wavenumber image. Norm-based metrics have been shown to improve robustness to global intensity variations while preserving chemically selective contrast.^40^ In the present study, vector normalization is considered particularly useful because it prevents the dominance of a small number of high-intensity pixels or even a single pixel.

Subsequent to normalization, PCA, NMF analysis, and k-means clustering were carried out on the set of chemical images to find sub-cellular structures. The pre-processing procedure emphasizes relative spectral differences rather than absolute absorbance values. While for this dataset no peak shifts were observed in the collected set of single-point spectra, such shifts do occur in other samples. In such cases, additional wavelengths can be added to the images to represent the peak shifts which would otherwise only appear as a change in intensity in the final images.

PCA^56^ has been widely used for dimensionality reduction and as an exploratory tool in hyperspectral imaging. The data is projected to orthogonal dimensions called principal components (PCs) oriented along the directions of the highest variance in the dataset. The PCs are a linear combination of the original variables, and the coefficients can be interpreted as weights that are stored in a loading matrix.^57^

Nonnegative matrix factorization (NMF)^58^ is a similar dimensionality reduction technique that decomposes a nonnegative sample matrix into nonnegative components. The representation of a data vector is obtained by adding the components (also called factors). NMF is therefore particularly useful for data of an additive nature, such as hyperspectral and multi-channel images.^59^

In contrast, k-means clustering, usually implemented *via* Lloyd's algorithm,^60,61^ is an unsupervised technique that clusters data so that the squared Euclidean distance between the row vector for any object and the centroid vector of its respective cluster is no larger than the distances to all other cluster centroids.

#### 2.3.3. Spectra

To substantiate the segmentation results obtained by PCA and NMF analysis, spectra were recorded at high-signal sites in the chemical images. Two spectra were averaged at each point of interest. All raw spectra were divided by the transmitted visible laser intensity for normalization. A more detailed description of the signal generation and background correction processes is given in Holub *et al.*^35^ To correct for differences in sample thickness, the mean spectra were normalized to the area under the Amide I band, more precisely to the region of 1585 cm^−1^ to 1720 cm^−1^.

The EC-QCL crossover transition between Chip 3 and Chip 4 resulted in a strong signal artifact in the region of 1265 cm^−1^ to 1355 cm^−1^, which had to be excluded from further analysis. In the below figures, the missing values were interpolated for better visualization. After calculating average spectra, these were smoothed by a first-order Savitzky–Golay filter with a window size of 12. The filter parameters aimed to preserve the main spectral features but remove excess noise.

## 3. Results

Single-wavenumber O-PTIR images of a H1299 cell from a different batch of the same parent cell line were recorded at several wavenumber settings representing the absorption bands outlined in 2.3.1. Spectra were recorded at points that were characterized by a marked signal in the 1100 cm^−1^ and/or 1660 cm^−1^ images.

**Fig. 2:**
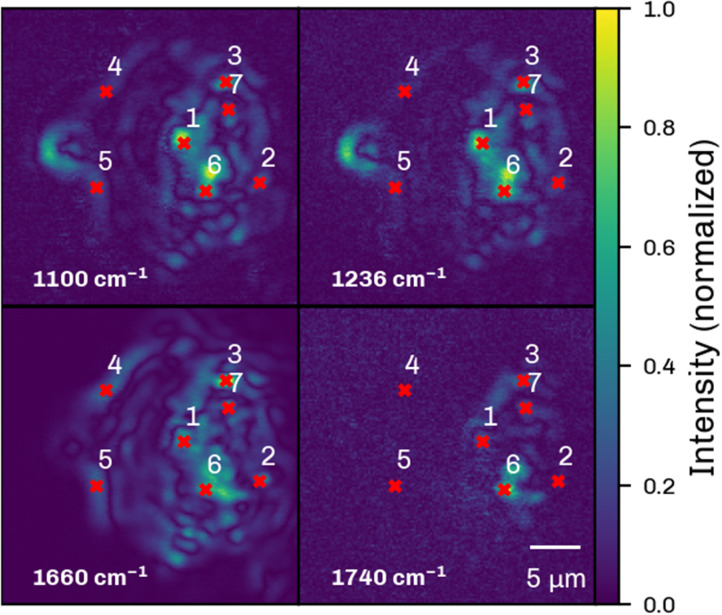
Normalized images of an H1299 cancer cell taken at 1100, 1236, 1660, and 1740 cm^−1^. The red markers (“x”) identify the locations at which spectra were recorded.

Fig. 2 shows 4 O-PTIR images recorded at the wavenumbers of 1100 cm^−1^, 1236 cm^−1^, 1660 cm^−1^, and 1740 cm^−1^. The image segmentation procedure described in the previous section was applied to the single-wavenumber images. Again, Sauvola's adaptive filter provided the hull that matched the cell shape most closely. All images were normalized prior to further analysis (see section 2.3.2).

CaF_2_ substrates are widely used in O-PTIR and related photothermal techniques. The sample preparation protocol applied in this study was optimized to minimize residual salts and other preparation-related artifacts, and no obvious crystalline residues were observed in the measured samples. To further reduce non-cellular background contributions, the spatial masking procedure described in section 2.3.1. was applied prior to downstream segmentation analysis. Following masking, PCA showed a stronger concentration of variance within the leading principal components (see Fig. S2), suggesting suppression of non-informative background variability.

In addition, the signal intensity of the spectra observed in this study is lower towards the periphery of the cell, suggesting that the signal originates from cellular material. Furthermore, spectral features are consistent with cell spectra previously obtained from a large number of samples analyzed under comparable experimental conditions.^35^ Together, these observations support the interpretation that the dominant spectral signatures originate primarily from cellular material.

Nevertheless, residual contributions from substrate non-uniformity or variations in the preparation process cannot be completely excluded.

### 3.1. PCA

The discrete-wavenumber images were subjected to PCA. As the wavenumbers had been selected from complementary spectral regions, the objective was to reveal spatial patterns within the masked dataset rather than a reduction of dimensionality. 4 PCs were chosen to build a composite image, with the aim of visualizing domains that may correspond to different cellular compartments. The percentages of total variance in the original dataset explained by the PCs are presented in Table 1. Using 4 PCs, only 1% of variance is unaccounted for by the model.

**Table 1:** PCs and explained variance (var.) for the PCA on the filtered images

PC	Explained var.
1	81%
2	10%
3	6%
4	2%

**Fig. 3:**
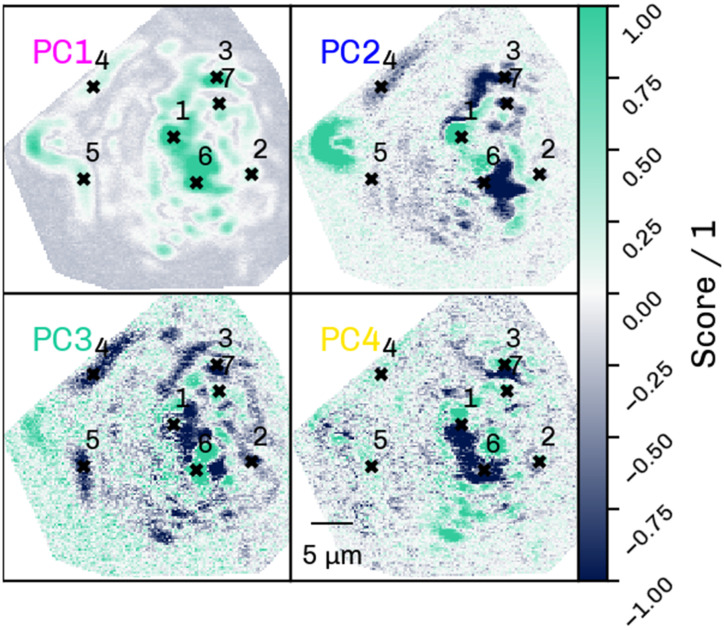
PCA score images corresponding to the 4 PCs. Scores can take positive (turquoise) and negative (black) values.

**Fig. 4:**
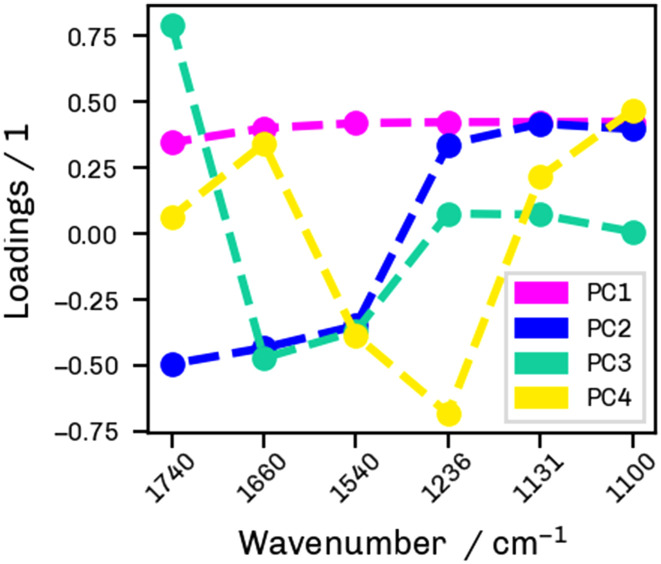
Loadings representing the contribution of the single-wavenumber signal to each PC.

Fig. 3 and 4 show the PCA.results. Fig. 3 presents the score plots, which illustrate the distribution of each PC over the sample area. Fig. 4 displays the factor loadings, which represent the contribution of the original variables (the wavenumbers) to each PC. It is important to note that PCA requires data to be mean-centered, resulting in scores and loadings that can be both positive and negative, with a negative score indicating a lower-than-average value of a PC.

In PC1, which explains 81% of the total variance in the dataset, all wavenumbers are nearly equally represented. PC1 should therefore closely match the area of the cell but not provide detailed information on possible sub-cellular structures. PC2 is negatively correlated with the bands of esterified lipids (1740 cm^−1^), Amide II (1540 cm^−1^) and Amide I (1660 cm^−1^), and positively correlated with the symmetric (1100 cm^−1^) and asymmetric (1236 cm^−1^) stretching vibrations of phosphates. PC3 can be mainly associated with esterified lipids, and PC4 is correlated with phosphate vibrations at 1100 cm^−1^ (positive correlation) and 1236 cm^−1^ (negative correlation) as well as the Amide I band.

The score images highlight two elongated regions near or within the cell membrane, which are characterized by pronounced positive scores of PC1 and high negative scores of PC2 and PC3. A region dominated by PC2 accentuates the bulb feature on the left side of the image. A dense, elliptical region potentially representing the nucleus is roughly outlined by high PC1 scores. The areas around Spectra 1, 3, 6 and 7 are not dominated by a single PC but rather show (positive and negative) correlations with all PCs.

### 3.2. NMF

**Fig. 5:**
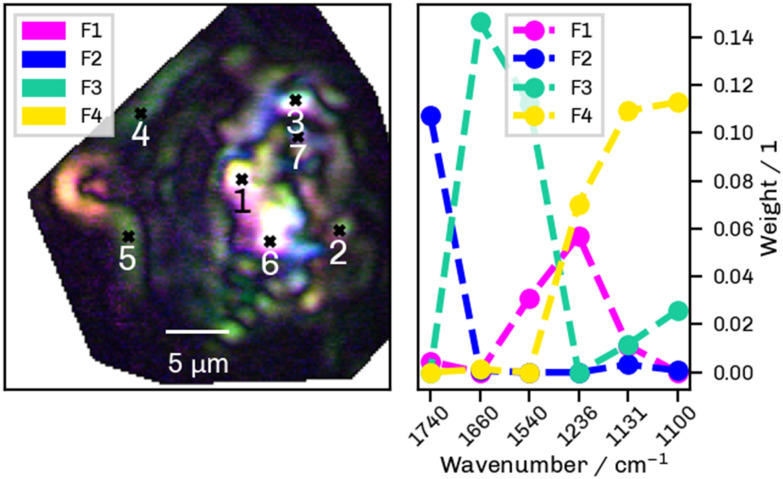
Left: Composite image visualizing the results of the NMF analysis (4 factors). Different colors indicate the relative contribution of the individual components. The numbered points indicate the coordinates at which spectra were collected. Right: Corresponding factor weights.

Analogous to the PCA settings, the NMF algorithm was configured to identify 4 factors. The composite image and the factor loadings (also called weights) are shown in Fig. 5. Please note that unlike the PCA matrices, the NMF scores and loadings have purely nonnegative values. Due to this nonnegativity, the factors can be visualized in a compact manner in a single multicolor overlay image, in which different colors indicate the relative contribution of the individual components.

The NMF factors correspond to more isolated wavenumber bands, with F1 representing the band at 1236 cm^−1^, F2 the band at 1740 cm^−1^, F3 the amide bands at 1540 cm^−1^ and 1660 cm^−1^, and F4 the phosphate vibrations at 1100 cm^−1^, 1131 cm^−1^, and 1236 cm^−1^. F1 and F4 therefore provide information on phosphate bond vibrations, while F2 visualizes regions in which there is a strong presence of esterified lipids. F3 represents the Amide I and Amide II bands, which are the most prominent vibrational bands in biological specimens. This factor is hence distributed fairly uniformly across the cell sample.

In the composite image, the background is strongly suppressed, and features emerge more clearly from the NMF approach than from the single-wavenumber images. The NMF visualization method emphasizes similar patterns as the PCA-based analysis: the F1 and F4 factors, which represent phosphate vibrations, are dominant in the bulb feature and a diffuse region that may be the cell nucleus. The elongated regions tentatively associated with the cell membrane are most prominently represented by the Amide I and II bands (F3) and the phosphate markers (F4), while the lipid band at 1740 cm^−1^ (F2) is limited to more isolated areas. White spots reveal areas of elevated NMF scores across the board.

It should be acknowledged that the wavenumbers were intentionally selected to align with established biological features, thereby pre-determining the chemical predominance observed in the final maps.

### 3.3. K-means clustering

**Fig. 6:**
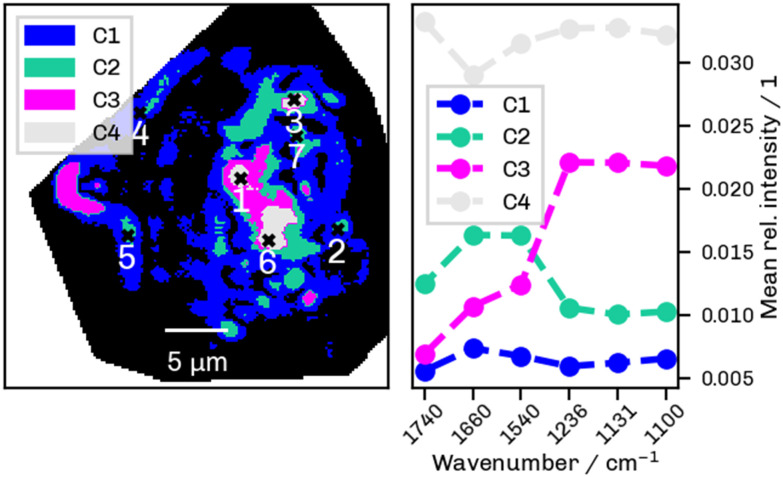
Left: Result of the k-means clustering process. The numbered points indicate the coordinates at which spectra were collected. Right: Intensities of the cluster centers. C0, which represents the background pixels, is not shown.

The k-means algorithm was set to generate 5 clusters (C0–C4), with C0 being the (black) background. As evident in Fig. 6, the result is in good agreement with the NMF analysis. The reason why 5 clusters were needed to match the output of 4 NMF factors on the same dataset is due to the underlying decomposition mechanism of the two algorithms. While NMF models data points as linear combinations of components, k-means is a rigid partitioning method that forces every point into one cluster.

The cluster centers, which are described in Fig. 6, provide insight into how the clusters were separated. For illustration purposes, the background cluster was omitted from the cluster center plot.

Similarly to F3, C2 has a strong contribution of the Amide I band, a low contribution of the esterified lipid band, and a low intensity at the phosphate-related bands. C2 comprises two elongated areas near the cell boundary as well as a large elliptical area. In contrast, the relative intensity of C3 is low for esterified lipids at 1740 cm^−1^ but very high at the phosphate bands of 1100 cm^−1^ and 1236 cm^−1^. This cluster represents the bulb feature and a smaller region within the elliptical area, which suggests a potential localization of nucleic-acid-rich structures. C1 has a moderate intensity in all bands, with the strongest contribution coming from the Amide I band. Like PC1, it covers most of the cell area. Lastly, in C4, the intensity is very high for all the aforementioned bands, with the relative contribution of the amide bands being slightly weaker. The regions dominated by C4 are the white clusters identified by the NMF algorithm.

### 3.4. Spectra

**Fig. 7:**
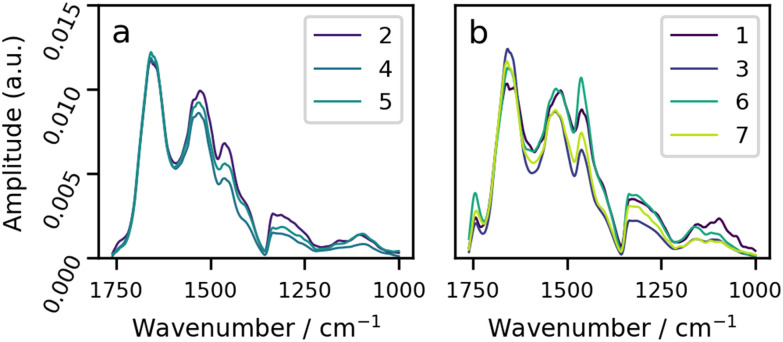
Spectra recorded at points of interest in the wavenumber region from 1000 cm^−1^ to 1760 cm^−1^. (a) Spectra associated with the membrane; (b) spectra associated with lipid-rich droplets.

Spectra were recorded at several points of interest (Fig. 7) to provide detailed information on potential sub-cellular domains and improve confidence in the results. Spectra 1, 3, 6, and 7 exhibit a marked esterified lipid band at 1740 cm^−1^ as well as a notably higher intensity in the band centered at 1465 cm^−1^, which can be attributed to CH_2_ and CH_3_ bending vibrations of proteins^51^ and phospholipids.^62^ These spectra were acquired in localized regions characterized by lipid-associated factors as indicated by all three algorithms. It is interesting to note that k-means clustering places Spectrum 7 in a different cluster, in which the esterified lipid vibrations are comparatively low.

Conversely, spectra 2, 4, and 5 were recorded near the structures associated with the cell membrane. The corresponding cell regions were characterized by a low esterified lipid signal along with low phosphate-related vibrations in the NMF and k-means clustering analyses. Two principal components, PC1 and PC3, describe the signal at these locations: PC1 is the celluar background, with almost equally distributed intensity values across all wavenumbers, while PC3 is characterized by a low esterified lipid component and strong intensities in the phosphate-related bands, which is more consistent with the composition of the cell membrane.^63^

## 4. Conclusions

This paper presents an easy-to-implement procedure for the pre-processing of single-wavenumber O-PTIR images using standard open-source libraries. Of the evaluated filters, the adaptive-threshold filter achieved the best result in selecting the area of interest. After background removal, three algorithms were tested to visualize sub-cellular domains in a H1299 cell sample: PCA, NMF, and k-means clustering.

Although ubiquitous in the analysis of hyperspectral data, PCA may not be the optimal choice for analyzing multi-wavenumber images. As PCA scores and loadings can have mixed signs, it is difficult to obtain a straightforward interpretation of the resulting image segmentation, and PCs may cancel each other out when combined in an overlay image. Furthermore, PCA is based on maximizing variance, which might not correspond directly to the most significant chemical or biological differences.

NMF was found to provide a more clear-cut distinction between the sample and the (remaining) background as well as between regions within the sample. Due to its non-negativity constraint, the weights and scores can be interpreted directly and may provide a physically and chemically more meaningful segmentation of chemical images.

The visual features obtained by k-means clustering were very similar to the NMF result, with the k-means approach yielding sharper boundaries between adjacent sample features. However, the chemical relevance of clusters is more difficult to establish, since cluster centroids represent an average of all features within a cluster and may not sufficiently point to differences between the clusters.

All three approaches were able to visualize small lipid-rich structures, which may suggest the presence of lipid droplets. Moreover, all methods highlighted elongated structures that could be part of the cell membrane and a high-signal area associated with the cell nucleus. These image segmentation results were corroborated by full spectral information obtained from individual point measurements.

Future work should explore the feasibility of the pre-processing approach presented and test its applicability to other biological samples.

## Author contributions

Elisabeth Holub: conceptualization, data curation, formal analysis, methodology, software, visualization, writing – original draft, writing – review & editing. Nikolaus Hondl: data curation, software, validation, writing – review & editing. Margaux Petay: methodology, writing – review & editing. Sarah Reindl: resources. Sophie Honeder: resources, writing – review & editing. Tamara Tomin: supervision, resources. Bernhard Lendl: supervision, resources, writing – review & editing. Georg Ramer: funding acquisition, project administration, resources, writing – review & editing.

## Conflicts of interest

There are no conflicts to declare.

## Supplementary Material

AN-151-D6AN00207B-s001

AN-151-D6AN00207B-s002

## Data Availability

The data underlying this study including raw data for hyperspectral images and spectra are openly available in Zenodo at https://doi.org/10.5281/zenodo.18711901. Supplementary information (SI): The SI includes a figure illustrating the effect of different filters on the convex hull mask, the SNRs of the single-wavenumber images, and the cumulative explained variance obtained from PCA. See DOI: https://doi.org/10.1039/d6an00207b.
