# Supplementary material for: Leveraging image processing techniques to visualize sub-cellular domains in optical photothermal infrared imaging
Source: Analyst. 2026 Jul 16. Online ahead of print. doi: 10.1039/d6an00207b (PMC13403074; doi:10.1039/d6an00207b)
Supplement: AN-OLF-D6AN00207B-s001 [file AN-OLF-D6AN00207B-s001.pdf]

Supplementary Information (SI) for Analyst.
This journal is © The Royal Society of Chemistry 2026

Supplementary information

Leveraging image processing techniques to visualize sub-cellular domains in optical photothermal infrared imaging

Elisabeth Holub[a,b], Nikolaus Hondl[a,b], Margaux Petay[a], Sarah Reindl[a], Sophie Honeder[a], Tamara Tomin[a], Bernhard Lendl[a] and Georg Ramer*[a,b]

[a] *TU Wien, Institute of Chemical Technologies and Analytics, Getreidemarkt 9, 1060 Wien, Austria*

[b] *TU Wien, Christian Doppler Laboratory for Advanced Mid-Infrared Laser Spectroscopy in (Bio-)process Analytics, Getreidemarkt 9, 1060 Wien, Austria. Tel: +43 1 58801 164151; E-mail: georg.ramer@tuwien.ac.at*

SI 1 Spectral ranges of MIRcat-QCT-z

	Chip 1	Chip 2	Chip 3	Chip 4
Range / cm^{-1}	2936-2347	1797-1347	1505–1195	1271–930

Table 1 Spectral ranges of the MIRcat-QCT-z

SI 2 Effect of different filters

Figure 1 shows the effect of the filters on the selected pixels and the resulting convex hull for each of the tested filters.

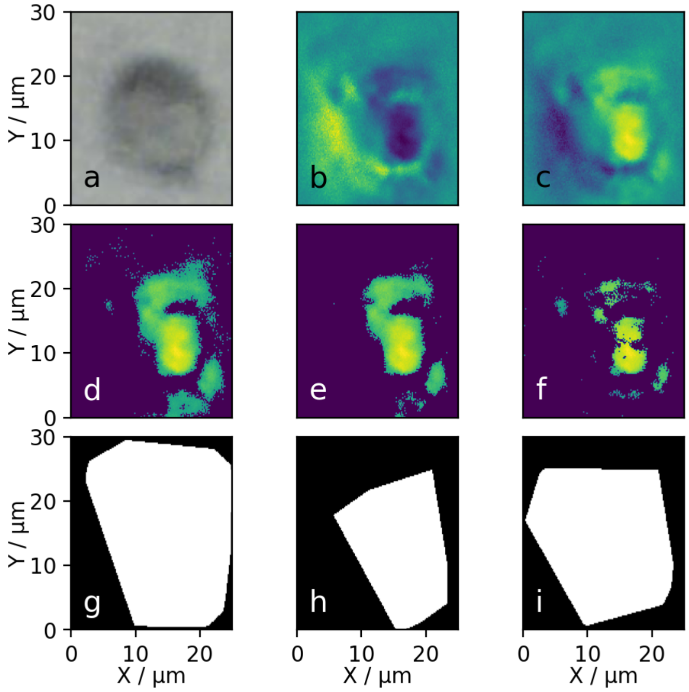

Figure 1 Threshold filters and resulting masks. a) brightfield image, b) transmission image, c) complement of the transmission image, d) and g): image after Otsu filtering and resulting mask; e) and h) image after Li filtering and resulting mask; f) and i): image after Sauvola filtering and resulting mask.

SI 3 Signal-to-noise ration of images

The signal-to-noise ratio (SNR) was defined as

$$SNR = \frac{Q_{90} - \mu_{noise}}{\sigma_{noise}}, \tag{1}$$

where Q_90 is the 90th percentile intensity in the in the region of interest, μ_{noise} is the mean intensity within a representative background region outside the ROI, and $sigma_{noise}$ is the standard deviation of the intensity in the background region. The 90th percentile provides robustness against single-pixel outliers and topography variations. The resulting SNRs of the single-wavenumber images prior to masking and segmentation are provided in Table

	1100 cm^{-1}	1131 cm^{-1}	1236 cm^{-1}	1540 cm^{-1}	1660 cm^{-1}	1740 cm^{-1}
SNR	12	24	7	50	134	4

Table 2 SNRs of the single-wavenumber images.

SI 4 Effect of preprocessing on explained variance

Figure 2 shows the cumulative explained variance obtained from principal component analysis (PCA) before and after the application of the spatial filter, that is the mask. After spatial filtering, there is a stronger concentration of variance within the leading principal components, indicating that masking reduced non-informative spatial variance.

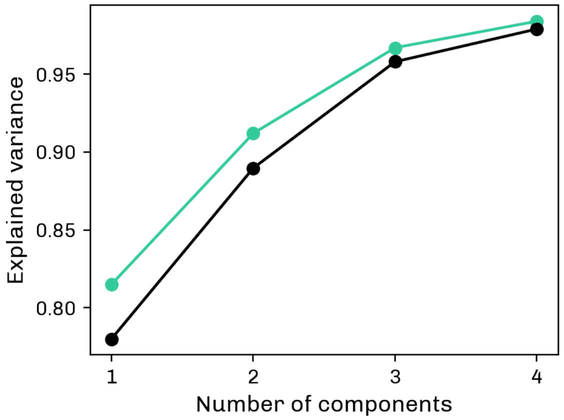

Figure 2 Cumulative explained variance for a PCA carried out before (black) and after (green) spatial pre-processing.
