# Supplementary figures and images for: Leveraging image processing techniques to visualize sub-cellular domains in optical photothermal infrared imaging
Source: Analyst. 2026 Jul 16. Online ahead of print. doi: 10.1039/d6an00207b (PMC13403074; doi:10.1039/d6an00207b)

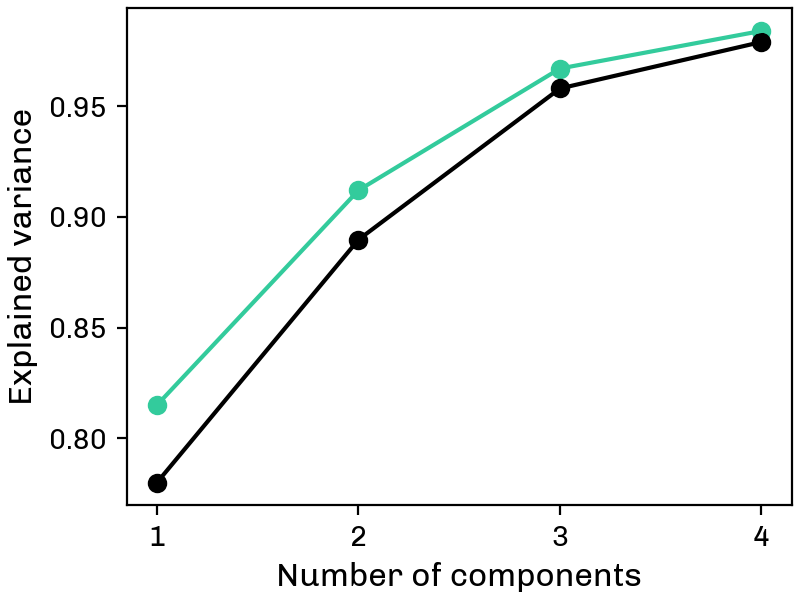

Supplement: AN-OLF-D6AN00207B-s002 [file AN-OLF-D6AN00207B-s002.zip › Figures/Variance.png]

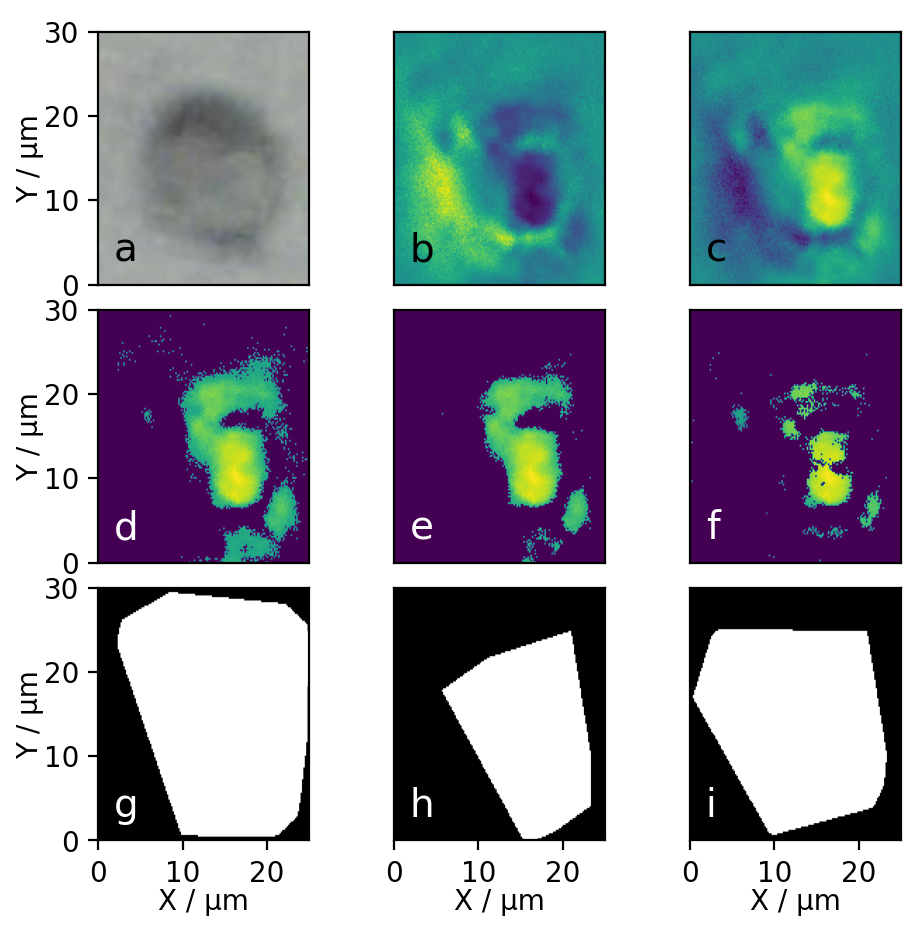

Supplement: AN-OLF-D6AN00207B-s002 [file AN-OLF-D6AN00207B-s002.zip › Figures/Filters2.png]
